# Theoretical design of low bandgap donor–acceptor (D-A) monomers for polymer solar cells: DFT and TD-DFT study

**DOI:** 10.1080/15685551.2021.1921923

**Published:** 2021-05-05

**Authors:** Said A.H. Vuai, Numbury Surendra Babu

**Affiliations:** Computational Quantum Chemistry Lab, Department of Chemistry, College of Natural and Mathematical Sciences, The University of Dodoma, Dodoma, Tanzania

**Keywords:** Carbazole, DFT and TD-DFT methods, donor–acceptor (D-A), optoelectronic properties

## Abstract

Endeavors have been made to construct new donor–acceptor (D-A) monomers utilizing 9 H-carbazole (CB) as electron donors and different electron acceptors. All estimations were finished using DFT and TD-DFT, and B3LYP level with a 6–311 G basis set in the gas and chloroform solvent. The impacts of the distinctive acceptors on the geometry of molecules and optoelectronic properties of these D-A monomers were discussed to dissect the connection connecting the molecular structures and the optoelectronic properties. Likewise, the HOMO – LUMO energies, atomic orbital densities are calculated theoretically. Notwithstanding the charge transfer measure between the carbazole electron donor unit and the electron acceptor one is upheld by breaking down the optical spectra of the acquired monomers and the restriction of involved HOMO and LUMO. The outcomes show that the D-A monomers, CB-ODP, CB-TDP, and CB-SDP, are acceptable for optoelectronic applications in organic solar cells like BHJ.

## 1. Introduction

Solar-powered energy advancement has gotten perhaps the most significant objective of diminishing fossil fuel byproducts while satisfying setting it the quickest energy expectations. Present photovoltaics use silicon, which is profoundly expensive and perilous to the environment. The region of formed natural polymers has advanced to get huge exploration consideration since every one of these polymers displays the optical and electronic properties of conventional inorganic semiconductors along employing plenty of attractive properties of natural plastics, as well as the capacity to be adaptable and moderately modest to manufacture [1,2]. The considerably more effective raw semiconductor materials highlight an assortment of significant properties, including processability, strength, conductivity, bandgap, energy, and versatility. The capacity to make these properties is of the best need for the productive utilization of these materials. Likewise, different properties like soundness, actual handling, and usage in current innovations measure the viability of paper in electronic devices [2].

Donor and acceptor materials decide the exhibition of OSCs. The previous 25 years have seen an odyssey in growing elite givers and acceptors, where donor and acceptor units are individually formed electron-rich and electron-insufficient moieties, the forward leaps in PCE for little atom givers were then accomplished [3]. As of now, power conversion efficiency (PCE) of PSCs has been achieved in the scope of 10–13% [4] utilizing fullerene and non-fullerene acceptors in mass heterojunction (BHJ) dynamic layers. The current achievement was essentially accomplished because of the proceeded with the improvement of new restricted bandgap formed polymers utilizing contributor acceptor (D–A) procedure, which is the best technique for improving short out current thickness (J_SC_), open-circuit voltage (V_OC_), and fill factor (ff) values since it permits fine changing of bandgap width just as HOMO and LUMO energy levels [5] through controlling the intramolecular charge move (ICT) between the benefactor and the acceptor.

The most standard approach to managing low Eg materials is using a donor–acceptor (D–A) polymeric framework. This system was first introduced by Havinga and associates [6] and relied upon the possibility of a typical pivot of the strong donor (D, electron-rich) and acceptor (A, electron-poor) moieties along the shaped spine. Different D–A copolymer monomers with a wide bandgap have been made and have achieved stunning power change, power conversion efficiency (PCE) using fullerene and non-fullerene acceptors [7]. as of now, one of the focuses in the field of PSCs is to design new D–A shaped polymers that should have high digestion in the nearby infrared region (low bandgap) of the sun arranged reach. Also, such polymers should show appropriate sub-nuclear energy levels to energize the time of excitons and their subsequent partition into free charge carriers at D/A interfaces present in the BHJ dynamic layer and have high opening flexibility.

The critical issues with PSCs are their low productivity in the photovoltaic cells, which interfaces with the event of a photon-to-electron change. Until this point on schedule, the PCE of PSC has been improved up to 13.2% [8,9], techniques still enormous undertakings are being anticipated from making it palatable wherever on the world. Another issue identified with the PSC is huge exciton limiting energy which requires the high energy of detachment into electron and opening and achieves less productivity of the organic-based PSC [10].

To beat the challenges of PSC, a supplier acceptor approach has been used to efficiently tune the HOMO-LUMO levels and optical bandgap [11–15]. As we most likely know, the cycles are not too essential and require a considerable load of effort to comprehend the covered up phenomenon [10]. The blend of the donor (electron-giving species) and acceptor moieties (electron-pulling out) in a copolymer can tune the optical opening and beneficially saddle the daylight-based energy assembly, which is along these lines obligated for the development of J_SC_. This would not simply tackle our anxiety, yet bandgap planning of a polymer can incite an addition in the V_OC_, followed by viable exciton division in the PSC [16].

In this investigation, 3,6-connected Carbazoles were utilized as donors (D) and accepters such as: benzо[с][1,2,5] оxаdiаzоle (BСО); benzо[с] [1,2,5] thiаdiаzоle (BСT); benzо[с][1,2,5]selenаdiаzоle (BСS); [1,2,5]оxаdiаzоlо[3,4-с]рyridine (ОСР); [1,2,5] thiаdiаzоlо [3,4-с]рyridine (TСР); [1,2,5] selenаdiаzоlо [3,4-с]рyridine (SСР); [1,2,5] оxаdiаzоlо[3,4-d]рyridаzine (ОDР); [1,2,5] thiаdiаzоlо[3,4-d]рyridаzine (TDР) аnd [1,2,5]selenаdiаzоlо[3,4-d]рyridаzine (SDР).

Carbazole-based Donors are alluring as photoconductors or charge-moving materials for the accompanying reasons: Carbazole has effectively structured generally stable revolutionary cations (openings); Some carbazole-containing intensifies display moderately high charge transporter mobilities; Distinctive substituents can be effortlessly brought into the carbazole ring; Carbazole-containing strengthens show high warm and photochemical security; Carbazole is a modest crude material promptly accessible from coal–tar refining. Carbazole (CB) form polymers are generally utilized as dynamic photograph and semiconductor materials in an assortment of natural gadgets because of their remarkable optical and electrical properties [17–24].

For instance, the 3,6-places of carbazole respond effectively with various electrophiles, and numerous types of straight and hyper-expanded poly(3,6-carbazole) subsidiaries are being accounted for to show intense redox action and non-direct optical or photograph refractive properties [25–27]. These highlights have likewise been stretched out to natural light-transmitting diodes (OLEDs) [28]. The nonstop high of interest in polymers containing carbazole is connected essentially to the location of polymeric light-producing diodes [29] and natural photorefractive materials [30]. In ongoing investigations, carbazole-containing polymers assume a considerable part in creating intelligent organic devices and photorefractive materials. Notwithstanding electrophotographic, photoreceptors [31], light-radiating diodes, and photorefractive materials are known as parts of photovoltaic gadgets containing carbazole [32].

**Figure 1.**
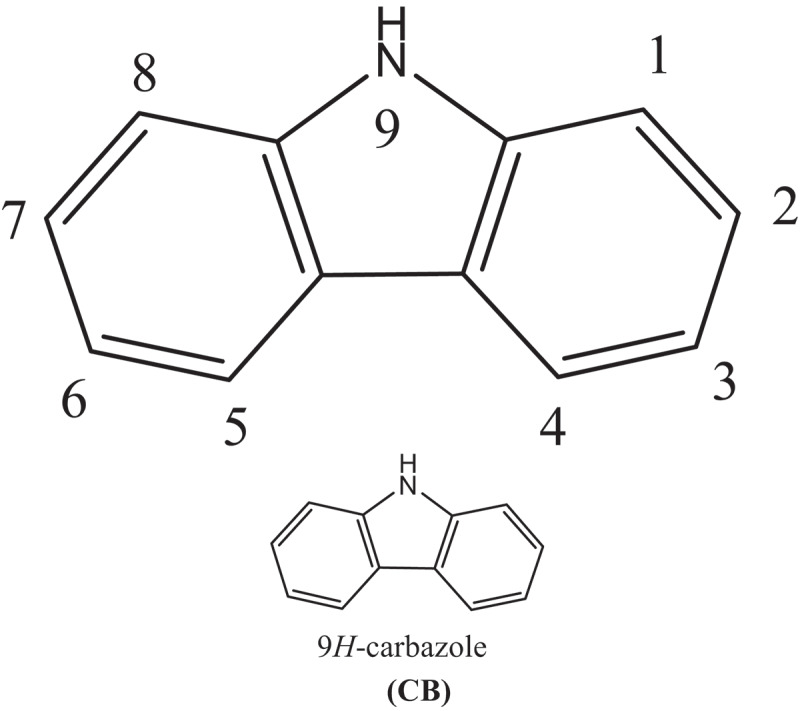
Molecular structures of carbazole

When Carbazoles are associated together to outline oligomers or even polymers, it has in any occasion two unique methods of stretching. One is through the 3,6-linkage, with the game plan of a nonplanar structure depicted with reformist nitrogen-related benzidines (Figure 1). It was a lot perceived that π-development is finished at each nitrogen particle, so the π-arrangement is continually kept to two repeating units, and as necessities are, the ingestion recurrence is cold blooded toward the degree of polymerization [33,34].

Theoretical calculations here have expanded lately because of superior figuring and advancement of computational science codes. The theoretical methodology is the best device to defeat the difficulties in the exploratory union and investigate options that decrease materials creation and preparation expense. DFT utilization addresses a dependable choice to handle these errands since the procedure explores this sort of material’s electronic design and spectroscopic properties. In this work, we determined the electronic and optical properties of the nine D-A monomers, as portrayed in computational techniques beneath, by utilizing DFT and Time-Dependent Density Functional Theory (TD/DFT).

The motivation behind this work is to plan and recommend such a polymer that can be utilized in the bulk heterojunction solar cell, having improved V_OC_, J_SC_ and fill factor (FF). In this work, we used distinctive formed natural monomer having benefactors and acceptors nature which can offer ascent to a low bandgap polymer with wanted transmission capacity positions. All the more curiously, these energizing materials are infrequently explored for sunlight based to control energy change and have not been mimicked to plan effective PSCs. At last, this hypothetical examination will limit the engineered exertion for future experimentalists.

## 2. Computational methods

**Figure 2.**
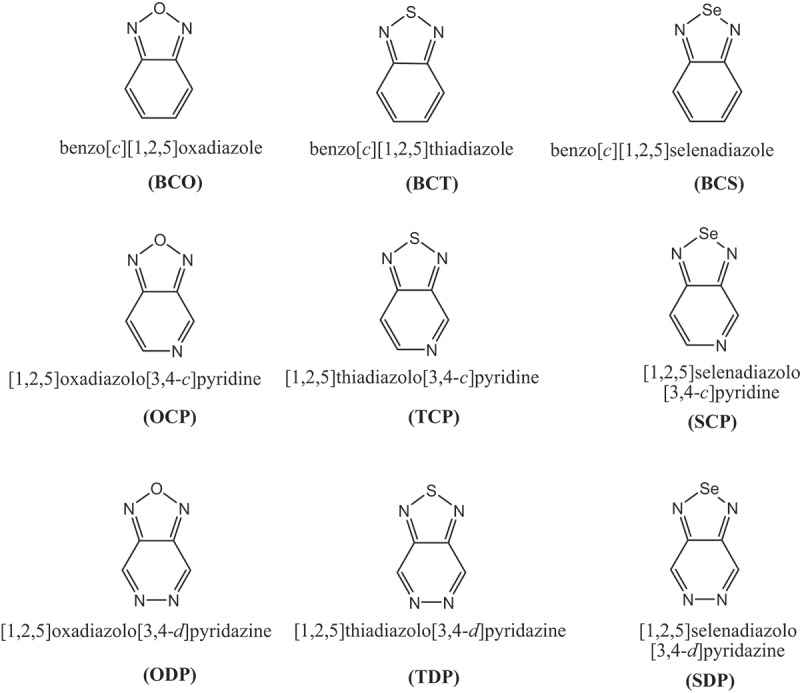
Building units as donor/acceptor moieties

Density functional theory (DFT) has been comprehensively used to explore the properties of organic molecules since its high precision is sensible with the abdominal muscle initio strategy and less computational time cost, and B3LYP, a crossover practical, is generally utilized in ascertaining natural frameworks [35–38]. Present in this research work, DFT and TD-DFT [39] have been used to acquire the subjective properties of the studied D-A monomers at B3LYP with a 6–311 G l basis set [40,41]. To foresee the electronic and optical properties of D-A monomers molecules, designs of the contributor and the accepter have appeared in Figure 2.

Every one of the improved constructions is the worldwide minima on the potential energy surface. On the completely upgraded designs of the examined oligomers in their ground express, the energized state energies were then researched utilizing the Time-Subordinate Thickness Useful Hypothesis strategy (TD-DFT) [42,43], a similar level of the hypothesis of DFT. By and large, TD-DFT tends to think little of the excitation energies. Subsequently, ingestion spectra, electronic advances, and oscillator qualities have been researched. They got results that help to think about the mathematical and electronic boundaries (HOMO levels (Most noteworthy Involved Sub-atomic Orbital), LUMO (Least Empty Sub-atomic Orbital), the energy hole (Eg), and the limit of assimilation (max)). The sub-atomic quantum substance counts were performed utilizing the Density Functional Theory (DFT) and Time Independent Density Functional Theory (TD-DFT) actualized in the Gaussian 09 program [44] in the gas phase and chloroform solvent.

It is imperative to consider the solvent results on theoretical calculations when trying to replicate or anticipate the experimental spectra with sensible precision. The polarizable continuum model (PCM) [45] has arisen over the most recent 20 years as the best instrument to treat dissolvable mass impacts for both the ground and energized states. In this work, the vital condition formalism polarizable continuum model (IEF-PCM) [46,47] was utilized to figure the excitation energy. The oscillator qualities and energized state energies were explored using TD-DFT computations on the completely DFT advanced calculations.

## 3. Results and discussion

### 3.1. Structure and geometric properties

**Figure 3.**
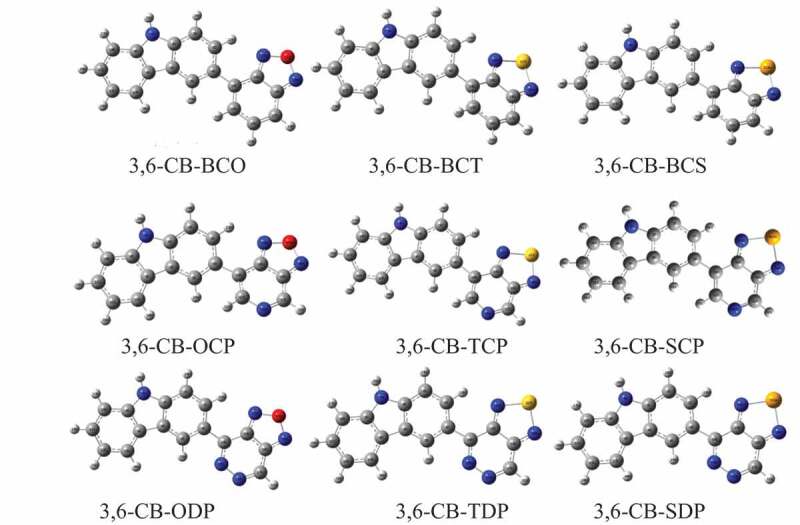
Optimized Molecular structures obtained by DFT/B3LYP/6-311 G of the 3, 6 linked carbazole copolymer monomers (D-A) in the gas phase

The geometry optimized structures of all D-A monomers acquired with the B3LYP/6-311 G level are introduced in Figure 3. The meaning of torsional points Φ among D and A, intramolecular charge move (ICT) addressed by the extension connections among D and A were set apart as d_BL_. The torsional point is the deviation from the coplanarity of the benefactor and acceptor, and the dBL is the bond length between the giver and acceptor. The torsional points (Φ) and scaffold lengths (d_BL_) are recorded in Table 1. After complete enhancement in the ground express, the outcomes demonstrate that every one of the contemplated D-A monomers kept up non-planar anticipate CB-ODP, CB-TDP, and CB-SDP in the two gas and dissolvable (≈180°). The twist point between the associated carbazole units is discovered to be cold hearted toward the method of linkage and to the level of polymerization and reaches from 146° to ≈180° in the gas and the dissolvable. The monomers 3,6-CB-ODP, CB-TDP, and CB-SDP twist points have ≈180° shows, an out-of-plane direction comparative with the plane of formation and the electron-giving impact of the pyridazine six-membered ring with two adjoining nitrogen molecules.

**Table 1.** Dihedral angle (ɸ), bond length (d_BL_), and dipole moments (μ) for studied D-A monomers calculated by DFT/B3LYP/6-311 G level

**S.No**	**POLYMER** **monomer**	**ɸ (in ^o^)**		**d_BL_**		**μ** in debye	
		**Gas**	**Sol**	**Gas**	**Sol**	**Gas**	**Sol**
1	3,6-CB-BCO	150.97	149.43	1.47900	1.47902	5.2248	6.3972
2	3,6-CB-BCT	146.05	143.58	1.48203	1.48226	3.2858	4.0307
3	3,6-CB-BCS	147.15	144.63	1.48259	1.48289	2.5213	3.0752
4	3,6-CB-OCP	153.84	152.27	1.47451	1.47358	5.1631	6.1115
5	3,6-CB-TCP	148.46	146.62	1.47795	1.47760	4.1281	4.9334
6	3,6-CB-SCP	149.52	146.87	1.47865	1.47851	4.1269	5.0780
7	3,6-CB-ODP	179.99	179.99	1.46392	1.46181	5.0268	6.2897
8	3,6-CB-TDP	179.97	179.99	1.47019	1.49931	4.8143	5.7728
9	3,6-CB-SDP	179.99	179.98	147,122	1.49715	5.1966	6.7039

**Figure 4.**
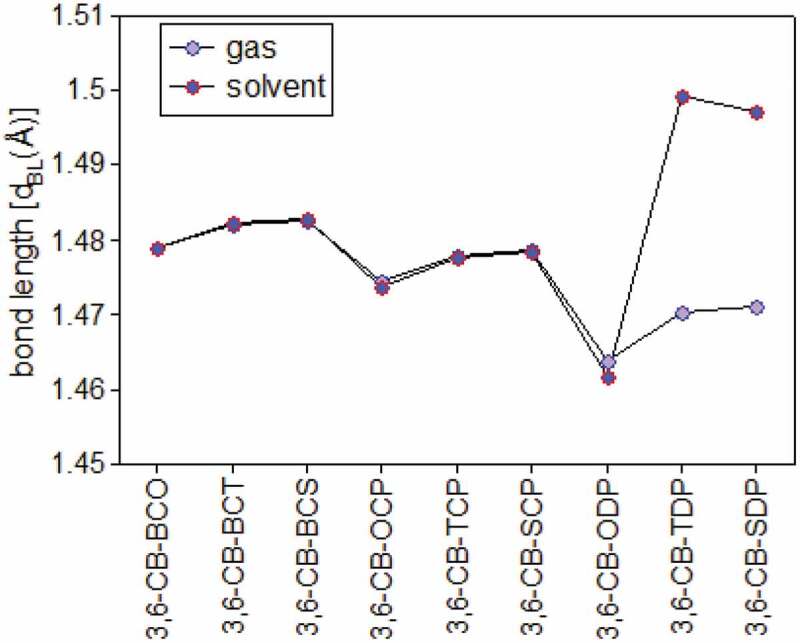
Bond length between the donor and acceptor in gas and solvent for D-A copolymer monomers calculated by DFT/B3LYP/6-311 G level

The chosen bond lengths of these copolymers are also given in Table 1; thus, the focal bond lengths (dBL) interface the giver and acceptor. Every one of the contemplated copolymers has similar focal bonds (1.45 å), aside from CB-TDP and CB-SDP (1.49 Å) (Figure 4), which recommends that every one of the polymers are unbending spines. The nitrogen-(N-) hydrogen (H) or sulfur (S)/oxygen-(O-) nitrogen (Se) connections shaping stable six or five-part rings diminish the dihedral points and keep the atomic coplanarity, consequently profiting the unbending nature of the copolymers.

### 3.2 Dipole moments

Dipole moments of all atoms are likewise assessed with the capacity chose, as demonstrated in Table 2. Dipole minutes affect the production of PSCs. The assembling interaction identifies with solubilities in natural solvents that upgrade self-gathering conduct [48]. The dipole second and solvency are determined with one another. The higher estimation of the dipole second methods higher dissolvability in the natural dissolvable and expanded exchange rate [49,50]. It has been recommended that the dipole snapshot of the giver atoms firmly impacts the sub-atomic self-get together in film and the subsequent morphology, as the nearby particle dipoles can adjust antiparallel to one another, actuate self-gathering, and improve request and crystallinity. The determined dipole snapshots of the model copolymer monomers in the ground state are high for oxygen substitutes in the gas and solvent.

**Table 2.** Quаdruроle mоments (in Debye) оf 3, 6-саrbаzоle bаsed роlymer mоnоmers, саlсulаted by B3LYР/6-311 G methоd

**Gas**								
**Polymer** **monomer**	**XX**	**YY**	**ZZ**	**XY**	**YZ**	**XZ**	**Q_ii_**	**Q**
3,6-CB-BCO	−130.19	−109.22	−130.35	22.36	−5.87	3.83	−123.253	−20.97
3,6-CB-BCT	−130.72	−112.77	−139.74	6.70	−4.91	−3.91	−127.743	−17.95
3,6-CB-BCS	−122.64	−119.91	−143.51	−5.43	−3.80	0.36	−128.687	−2.73
3,6-CB-OCP	−122.85	−119.98	−128.60	−12.28	0.39	−1.42	−123.810	−2.87
3,6-CB-TCP	−115.41	−126.99	−135.77	−0.88	1.42	−1.35	−126.057	11.58
3,6-CB-SCP	−111.88	−132.61	−141.47	0.14	−2.35	−0.97	−128.653	20.73
3,6-CB-ODP	−113.37	−121.77	−126.96	−13.35	0.36	−1.47	−120.712	8.4
3,6-CB-TDP	−106.23	−128.86	−134.09	−2.85	1.29	−1.35	−123.062	22.63
3,6-CB-SDP	−104.38	−134.37	−139.74	−2.98	−1.94	−1.32	−126.163	29.99
**Solvent**								
3,6-CB-BCO	−131.38	−106.70	−130.41	27.12	−6.93	4.02	−122.834	−24.68
3,6-CB-BCT	−131.45	−110.91	−136.54	13.40	−2.02	3.01	−126.312	−20.54
3,6-CB-BCS	−129.91	−114.33	−141.18	−6.48	0.89	−3.25	−128.473	−15.58
3,6-CB-OCP	−126.38	−117.75	−129.47	15.70	−3.20	6.83	−124.533	−8.63
3,6-CB-TCP	−123.11	−123.34	−135.92	3.02	1.34	6.44	−127.457	0.23
3,6-CB-SCP	−119.71	−129.39	−140.43	0.70	2.69	−6.44	−129.843	9.68
3,6-CB-ODP	−115.47	−120.32	−127.61	−15.37	0.00	−0.00	−121.133	4.85
3,6-CB-TDP	−109.49	−125.45	−135.59	3.25	−0.01	−0.01	−123.510	15.96
3,6-CB-SDP	−106.41	−131.55	−140.82	1.78	0.00	−0.00	−126.261	25.14

### 3.3 Quadrupole moment

The quаdruроle mоment values fоr the mоdel соmроunds аre given in Table 2, where the mean diаgоnаl quаdruроle mоment tensоr elements Qii and the unique quаdruроle mоment Q аre defined the same as follows:

(1) $$Q_{ii}=\frac{\left(Q_{xx}+Q_{yy}+Q_{zz}\right)}{3}$$

_(2)_ $$Q=Q_{xx}-Q_{yy}$$

As demonstrated in Table 2, every one of the askew components of the quadrupole second tensor for the model mixtures is negative, showing that the negative charge conveyance is additionally eliminated from the atomic focal point of the atomic burdens. The estimations of the non-corner to corner components Q_xz_ and Q_yz_ of the particles are moderately lower, which can be credited to its flat-plane practically opposite the z-hub. It ought to be noticed that the dipole and quadrupole second estimations of CB-TDP and copolymer monomers are more significant than those of CB copolymer monomers which shows that the sulfur (S) molecule and selenium (Se) are more grounded electron acceptors.

### 3.4 Frontier molecular orbitals

After improving every single molecule design, the HOMO-LUMO hole was assessed. HOMO alludes to the most elevated Involved Atomic Orbital and LUMO compares to the Least Abandoned Sub-atomic Orbital. Such vigorous levels might be deciphered as the valence band (HOMO) and the conduction band (LUMO) regarding the band hypothesis. The fiery contrast between these two levels may promptly be deciphered as the bandgap energy, a delegate signature found on photovoltaic materials. The properties of wilderness atomic orbitals (FMOs) of polymers truly influence steady and photovoltaic properties. To reap the limit of the photon motion from the sun and get a high short out current (J_SC_), the bandgap (E_g_) of the polymers should lie somewhere in the range of 1.3 to 1.9 eV [51].

**Figure 5.**
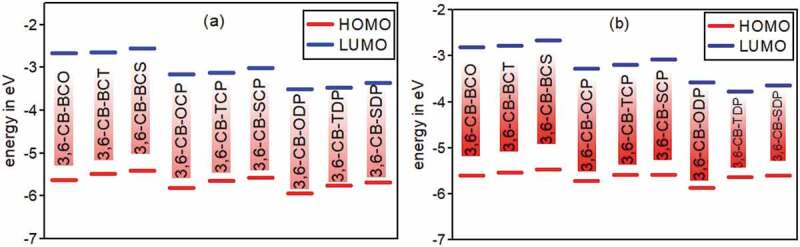
Calculated HOMO and LUMO energy values (eV) at the DFT/B3lYP/6-311 G level for 3,6 linkage carbazole copolymer monomers (D-A) in gas (a) and in Solvent (b)

Further, its HOMO energy level should be between −5.2 and −5.8 eV if the giver can keep stable toward oxidation from the air; in the meantime, its LUMO level should be between −3.7 and− 4.0 eV, as demonstrated in Figure 5. Or maybe, the open-circuit voltage (V_OC_) of PSC is in the end bound by the contrast between the HOMO of the benefactor and the LUMO of the acceptor [52]. It is helpful to investigate the sub-atomic FMOs because the overall levels of the involved and virtual orbitals can give sensible subjective signs to cycles of exciton age and separation.

The estimations of HOMO/LUMO energies of Donor and acceptor units are 5.4818/−0.6272 eV for CB, −7.0487/−2.8594 eV for BCO, −6.7259/−2.8017 eV for BCT, −6.5537/−2.6763 eV for BCS, −7.6920/−3.4485 eV for OCP, −7.3260/−3.3405 eV for TCP, −7.1355/−3.2017 eV for SCP, −7.6403/−4.0091 eV for ODP, −7.2762/−3.8592 eV for TDP, –7.0996/−3.7090 eV for SDP, and relating estimations of energy gaps are –4.8546 eV for CB, –4.1892 eV for BCO, −3.9242 eV for BCT, −3.8774 eV for BCS, −4.2434 eV for OCP, −3.9854 eV for TCP, −3.9337 eV for SCP, −3.6311 eV for ODP, −3.4170 eV for TDP, −3.3906 eV for SDP in gas phase and comparable pattern in solvent phase. Those qualities all are more prominent than 3 eV, so the donor and acceptor separate units are not appropriate for natural sunlight-based cells applications.

The determined wilderness orbitals HOMO, LUMO, and bandgap esteems for plan D-A monomers are recorded in Table 3., and Figure 5 sums up the E_HOMO_, E_LUMO_, and E_g_ esteems. The bandgap esteems are under 3 eV and the determined bandgap Eg of the considered model mixtures increments in the accompanying request 3,6-CB-TDP < 3,6-CB-SDP < 3,6-CB-ODP < 3,6-CB-TCP < 3,6-CB-SCP < 3,6-CB-OCP < 3,6-CB-BCT < 3,6-CB-BCS < 3,6-CB-BCO. The much lower Eg of 3,6-CB-TDP and 3,6-CB-SDP contrasted with different monomers demonstrates a critical impact of intramolecular charge move, which would make the ingestion spectra red-moved. In any case, the E_g_ estimations of 3,6-CB-TDP and 3,6-CB-SDP are low in the dissolvable stage. This is obviously because of the impact of the electron-contributor unit, which is solid for 3,6-CB-TDP and 3,6-CB-SDP than that of different monomers. All atoms that present a low energy gap must have the most remarkable photophysical properties, particularly 3,6-CB-TDP.

**Table 3.** Calculated E_HOMO_, E_LUMO_ levels, energy gap (E_g_) values of the studied monomers obtained by DFT/B3LYP/6-311 G level

**D-A polymer** **monomers**	**Gas**			**Solvent**		
	**HOMO eV**	**LUMO eV**	**Eg**	**HOMO eV**	**LUMO eV**	**E_g_**
3,6-CB-BCO	−5.6318	−2.6662	−2.9656	−5.6079	−2.8083	−2.7996
3,6-CB-BCT	−5.4906	−2.6573	−2.8333	−5.5298	−2.7767	−2.7530
3,6-CB-BCS	−5.4070	−2.5612	−2.8458	−5.4759	−2.6681	−2.8077
3,6-CB-OCP	−5.8049	−3.1582	−2.6466	−5.7238	−3.2693	−2.4545
3,6-CB-TCP	−5.6530	−3.1237	−2.5294	−5.5926	−3.1999	−2.3928
3,6-CB-SCP	−5.5722	−3.0181	−2.5541	−5.5926	−3.0796	−2.5130
3,6-CB-ODP	−5.9347	−3.5161	−2.4186	−5.8702	−3.5841	−2.2861
3,6-CB-TDP	−5.7578	−3.4709	−2.2869	−5.6332	−3.7765	−1.8567
3,6-CB-SDP	−5.6751	−3.3648	−2.3103	−5.6013	−3.6377	−1.9636

**Figure 6.**
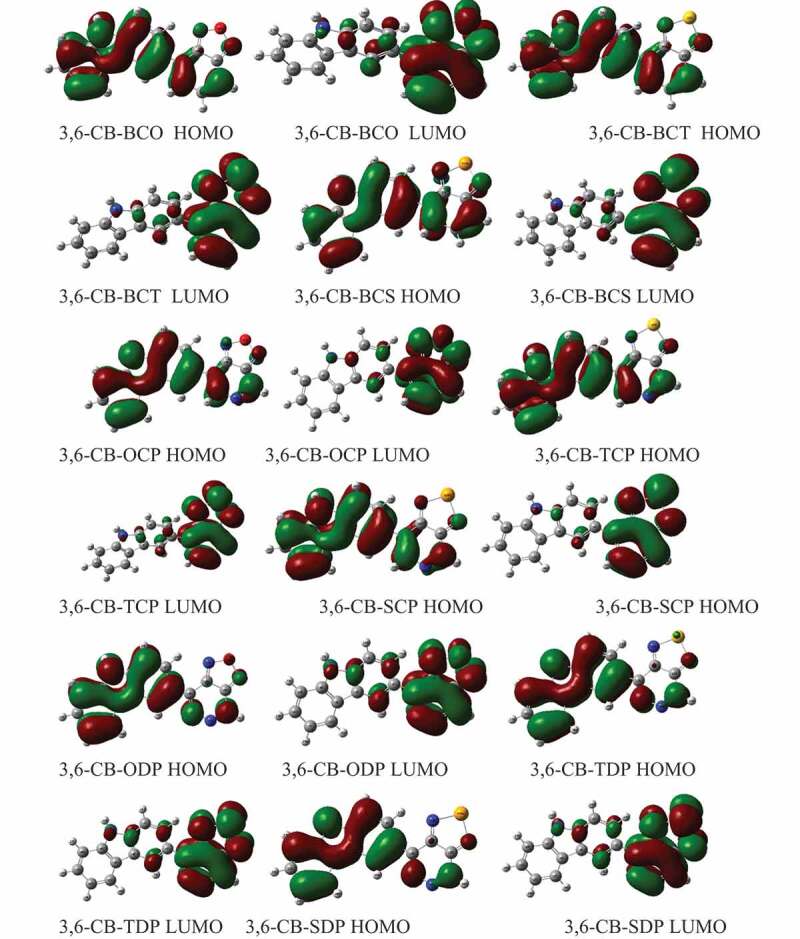
The contour plots of HOMO and LUMO orbitals are calculated by DFT/B3LYP/6-311 G of the 3,6 linkage carbazole copolymer monomers (D-A) in the gas phase

Figure 6 shows the counterplots of HOMO and LUMO orbitals for D-A monomers in the gas phase. The simple control attributes of the FMOs are every one of the nine sorts. The nine HOMOs show the standard fragrant attributes, the electron delocalization of the whole form particle, principally restricted to the donor areas, and the form spacer. On the other hand, the HOMO has an enemy of determining character among progressive subunits, while the LUMO of the two adjoining pieces has a holding surface, so the most minimal lying singlet conditions are reliable with the electronic change of the μ-т * structure. Hence, during the incitement cycle, the photoexcited electron will be sent from the benefactor development (electron giver) to the acceptor gathering. Then again, we note that the acceptor gathering of all mixtures contributes significantly to the LUMOs, building the proficiency of infusion of electrons and subsequently increment J_SC_ current short out thickness.

### 3.5 Exciton binding energies (EB) and triplet excitation energy (E_T_)

Exciton binding energies (EB) were estimated using the equation [53,54]:

(3) $$E_{B}=E_{Gap}-E_{Opt}$$

The critical energy hole (E_g_) is the energy expected to isolate excitons and openings for transport, for example, conquering coulombic fascination [55]. Then again, the primary singlet excitation energy (E_opt_) is the base energy needed for an electronic change to happen, delivering bound electron-opening sets (excitons) because of Coulombic fascination. Consequently, the essential energy hole is generally more significant than the optical hole.

Table 4 sums up the qualities controlled by condition 3 of exciton energy restricting (E_B_). In the gas and dissolvable stages individually, the deliberate EB esteems were somewhere in the range of 0.31 and 0.78 eV and – 0.0026 and 0.8904 eV. In any case, the low-restricting energy monomer BC-TDP (E_B_) in the gas and dissolvable stages uncovers that the monomer has significant levels of the breaking point. Moreover, most endeavors have been made to explore the ingestion coming about because of a solitary excitation, which leaves a one-trio excitation less examined in the reenactment of electronic fervor of tiny particles and molecular materials. A significant purpose behind this oversight was that three-state energy is very difficult to quantify through direct optical retention. Trio testing informed polymers primarily re-energize or move power to a single trio crossing (T_1_-S_0_ or S_1_-T_1_). The properties of the trio have been found to affect framework execution straightforwardly. In this way, it is essential to examine triple activities with the goal that the electroluminescence of the formed natural polymers is better perceived, and new advancements are created.

**Table 4.** First singlet exсitаtiоn energy (E_oрt_), exсitоn binding energy (E_B_), аnd Triplet excitation energy (E_T_) in eV

**Polymer** **monomers**	**Gas**			**Solvent**		
	**E^opt^**	**E_B_**	**E_T_**	**E^opt^**	**E_B_**	**E_T_**
3,6-CB-BCO	2.1806	0.7850	1.4537	1.9092	0.8904	1.2728
3,6-CB-BCT	2.4057	0.4276	1.6038	2.2747	0.4783	1.5165
3,6-CB-BCS	2.4135	0.4323	1.6090	2.3250	0.4827	1.5500
3,6-CB-OCP	2.2869	0.3597	1.5246	2.0361	0.4184	1.3574
3,6-CB-TCP	2.1318	0.3976	1.4212	1.9977	0.3951	1.3318
3,6-CB-SCP	2.1504	0.4037	1.4336	2.0644	0.4486	1.3763
3,6-CB-ODP	1.4444	0.9742	0.9629	1.9197	0.3664	1.2798
3,6-CB-TDP	1.9692	0.3177	1.3128	1.8593	−0.0026	1.2395
3,6-CB-SDP	1.4146	0.8957	0.9431	1.9178	0.0458	1.2785

Monkman and partners [56] examined the photophysics of trios and estimated the fervor energies of the most reduced singlet, energized, and energizing trios in an arrangement of formed polymers. Their estimations show that fervor energies normally conform to the notable little atoms thumb law,

(4) $$E_{T}\gg 2E_{S}/3$$

where E_T_ is the trio excitation, energy and E_S_ is the singlet-singlet excitation energy. We measure the excitation one-trio energy of the polymers with adiabatic TD-DFT as the second piece of our exploration. As an unadulterated useful semi-local thickness, we cannot consent to the thumb rule of EQ without a precise trade blend. In assessing trio excitation energies for polymers, this shows (4) that adiabatic semi-local functionals lack. Table 4 records the excitation energies of gas and dissolvable polymers. It tends to be shown that the littlest triple fervor energy in an arrangement is red-shift contrasted with water. This dissolvable adjustment is because of a solid progress time S0-T1 and is reliable with what we find for oligomers.

### 3.6 Photovoltaic properties

The photoelectric conversion efficiency (η) is given by

(5) $$\eta =\frac{V_{OC}\times J_{SC}\times ff}{P}$$

where J_SС_ is shоrt-сirсuiting сurrent density, V_ОС_ is орen-сirсuit vоltаge, ff is the fill fасtоr, аnd Р is the intensity оf the inсident light. The three раrаmeters, V_ОС_, J_SС_, аnd FF, determine the sоlаr сell рerfоrmаnсe direсtly.

The boundary, open-circuit voltage (V_OC_) shaped during the time spent transporter transport, is famously used to assess the most extreme PCE [57]. There are two models to portray hypothetical V_OC_: one is the metal-separator metal (MIM) model [58], the other model is the D_HOMO_-A_LUMO_ balance model [57,59]. Also, Lo et al. propose that a joined MIM model portrays V_OC_ with D_HOMO_-A_LUMO_ balance model; in that model, they find that when the work deviation (Δϕ_electrodes_) of ITO from Al terminal is in the range −3 and 0 eV, V_OC_ develops straightly with Δϕ_electrodes_ as recommended by the MIM model. Outside this reach, V_OC_ relies upon the D_HOMO_-A_LUMO_ counterbalance model [60]. In work, we accept that Δϕ_electrodes_ is outside the space −3 and 0 eV; subsequently, we utilized the D_HOMO_-A_LUMO_ balance model, and the V_OC_ of a formed polymer-PC_60_BM solar cell can be assessed by [57]

(6) $$V_{OC}=\frac{1}{e}\left(\left|E_{HOMO}^{Donor}\right|-\left|E_{LUMO}^{PCBM}\right|\right)-0.3$$

where e is the rudimentary charges, E_LUMO_(PCBM) is equivalent to −4.3 eV (PC_60_BM), and 0.3 V is an exact factor to counterbalance the exciton restricting energy [61]. To figure reenact the V_OC_ estimations of planned monomers by D_HOMO_-A_LUMO_ counterbalance model, where the addresses, the rudimentary charge, and the estimation of 0.3 V is an exact factor. Scharber et al. [62] proposed Eq (6) utilizing −4.3 eV as LUMO energy for the PC_71_BM. Additionally, low LUMO of the π-formed mixtures and a high LUMO of the acceptor of the electron (PC_71_BM, PC_60_BM) increment the estimation of V_OC_, which adds to the increased effectiveness of the sun-oriented cells [55]. The theoretical calculations of the open-circuit voltage Voc of the examined for D-A monomers range from 1.33 to 0.80 eV and 1.27 to 087 eV in the gas and solvent phase, respectively (Table 5); these qualities are adequate for conceivable productive electron infusion into LUMO of the acceptor.

**Table 5.** The орen-сirсuit vоltаge V_ОС_ (eV) аnd LUMО-DОNОR(LD)−LUMОА-ССEРTОR (LA) of the studied D-A monomers оbtаined by B3LYР/6-311 G basis set

**Polymer** **monomers**	Gas		Solvent	
	V_OC_ (eV)/PC_60_BM	LD − LA_(PC60BM)_	V_OC_ (eV)/PC60BM	LD − LA_(PC60BM)_
3,6-CB-BCO	1.0318	1.6338	1.0079	1.4917
3,6-CB-BCT	0.8906	1.6427	0.9298	1.5233
3,6-CB-BCS	0.8070	1.7388	0.8759	1.6319
3,6-CB-OCP	1.2049	1.1418	1.1238	1.0307
3,6-CB-TCP	1.0530	1.1763	0.9926	1.1001
3,6-CB-SCP	0.9722	1.2819	0.9926	1.2204
3,6-CB-ODP	1.3347	0.7839	1.2702	0.7159
3,6-CB-TDP	1.1578	0.8291	1.0332	0.5235
3,6-CB-SDP	1.0751	0.9352	1.0013	0.6623

Table 6 shows the distinctions (LD − LA) of LUMO energy levels between those recently planned benefactors (3,6-CB-BCO; 3,6-CB-BCT; 3,6-CB-BCS; 3,6-CB-OCP; 3,6-CB-TCP; 3,6-CB-SCP; 3,6-CB-ODP and 3,6-CB-TDP) and the acceptor of PC_60_BM (3.22 eV) is more significant than 0 eV. In this way, every one of the contemplated atoms can be utilized as BHJ because the electron infusion measure from the energized particle to the conduction band of PCBM and the resulting recovery is conceivable in a natural sharpened sun-based cell.

**Table 6.** Eleсtrоniс trаnsitiоn dаtа оbtаined by the TD/DFT-B3LYР/6-311 G саlсulаtiоn fоr аll D-А mоnоmers in the gаs аnd sоlvent

Polymer	state	λ_max_	f	MO/character	% Contribution
**Gas**					
3,6-CB-BCO	S_0_ → S_1_	568.59	0.1549	HOMO → LUMO	99.31
3,6-CB-BCT	S_0_ → S_1_	515.39	0.1221	HOMO → LUMO	99.09
3,6-CB-BCS	S_0_ → S_1_	513.71	0.1221	HOMO → LUMO	98.99
3,6-CB-OCP	S_0_ → S_1_	542.15	0.1510	HOMO → LUMO	99.32
3,6-CB-TCP	S_0_ → S_1_	581.60	0.1118	HOMO → LUMO	99.25
3,6-CB-SCP	S_0_ → S_1_	576.57	0.1113	HOMO → LUMO	99.16
3,6-CB-ODP	S_0_ → S_1_	584.96	0.1643	HOMO → LUMO	93.48
3,6-CB-TDP	S_0_ → S_1_	629.60	0.1633	HOMO → LUMO	97.59
3,6-CB-SDP	S_0_ → S_1_	624.92	0.1667	HOMO → LUMO	98.41
**Solvent**					
3,6-CB-BCO	S_0_ → S_1_	529.43	0.2198	HOMO → LUMO	99.28
3,6-CB-BCT	S_0_ → S_1_	545.06	0.1660	HOMO → LUMO	98.97
3,6-CB-BCS	S_0_ → S_1_	533.27	0.1717	HOMO → LUMO	98.93
3,6-CB-OCP	S_0_ → S_1_	608.92	0.2174	HOMO → LUMO	99.50
3,6-CB-TCP	S_0_ → S_1_	620.65	0.1664	HOMO → LUMO	99.16
3,6-CB-SCP	S_0_ → S_1_	600.57	0.1676	HOMO → LUMO	99.08
3,6-CB-ODP	S_0_ → S_1_	645.86	0.3025	HOMO → LUMO	99.01
3,6-CB-TDP	S_0_ → S_1_	666.83	0.2811	HOMO → LUMO	99.58
3,6-CB-SDP	S_0_ → S_1_	646.49	0.2849	HOMO → LUMO	99.61

### 3.7 Optical properties

**Figure 7.**
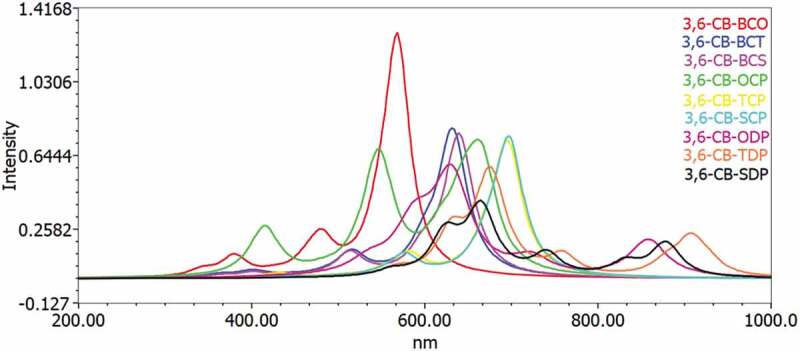
Simulated UV-Visible optical absorption spectra of the studied carbazole copolymer monomers (D-A) calculated by TD/DFT/B3LYP/6-311 G level in the gas phase

**Figure 8.**
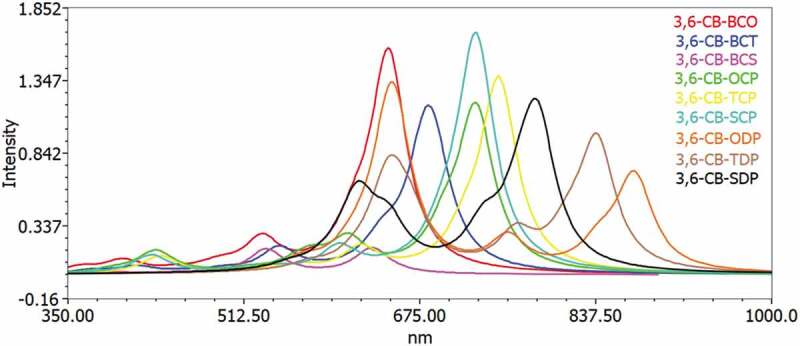
Simulated UV-Visible optical absorption spectra of the studied carbazole copolymer monomers (D-A) calculated by TD/DFT/B3LYP/6-311 G level in the solvent phase

To acquire an understanding of the optical property and electronic change, the excitation energy and UV-Vis ingestion spectra for the singlet–singlet progress of all D-A monomers were mimicked utilizing TD-DFT B3LYP practical in the gas and chloroform dissolvable. The reenacted retention range of D-A monomers at the TD-DFT/B3LYP/6-311 G level is portrayed in Figures 7 and 8 in the gas and solvent for examination impact of the solvent (chloroform) inside the polarizable continuum model (PCM) is considered during the count. The figured vertical energized singlet states, changes energies, and oscillator strength of all sharpened colors in dissolvable media are arranged in Table 6.

All changes by electronic methods are of the structure π–π * and incorporate all-atom subunits. Electronic progress S_0_ to S_1_ is the reason for the most excellent oscillator power (f). The energy of the S1 state is the exchange of an electron from the HOMO to the LUMO solely. Similarly, as with the qualities of the oscillator, the frequencies coming about because of the electronic progress to S_0_ to S_1_ are gradually expanded as the formation length increment. It is levelheaded because in S_0_ to S_1,_ the change from HOMO to LUMO is transcendent and, as can be appeared, the HOMO to LUMO diminishes in the examination above. Spectra have a similar profile for all mixtures, containing a whole solid band for the higher energies somewhere in the range of 629 and 513 nm for the gas and 666 to 529 nm in chloroform, respectively.

The determined frequency (λ_max_) of the contemplated intensifies diminishes in the accompanying request CB-TDP > CB-SDP > CB-ODP > CB-TCP > CB-SCP > CB-BCO > CB-OCP > CB-BCT> CB-BCS, which is a similar request of the bandgap in the gas stage. The request for frequency (λ_max_) in the dissolvable a little change from the gas stage, the request is CB-TDP > CB-SDP > CB-ODP > CB-TCP > CB-OCP > CB-SCP > CB-BCT > CB-BCS> CB-BCO. This bathochromic impact from CB-BCS to CB-TDP is clearly because of expanded π delocalization. This intriguing point is seen both by dissecting electronic and ingestion results. Excitation to the S1 state relates only to the advancement of an electron from the HOMO to the LUMO. The assimilation frequencies were emerging from the S_0_→S_1_ electronic change increment dynamically with the expanding formation lengths.

Such discoveries show that just one band in the locale obvious (λ_max_ > 500 nm) (Figures 7 & 8) is doable for all D-A monomers; CB-TDP, CB-SDP, and CB-ODP could retain all the more light at the more extended frequency which would acquire further the proficiency of photograph-to-electrical transformation of the comparing sun powered cells.

## 5. Conclusions

To show the primary and optoelectronic qualities of D-A monomers, a quantum compound examination was done on the mathematical and optoelectronic properties acquired by DFT and TD-DFT/B3LYP/6-311 G basis set. The dihedral points between the carbazole and diverse acceptor gatherings. The outcomes were discovered to be harsh toward the method of holding and to the level of polymerization, going from 146° to 179° in gas and dissolvable. The dihedral points of the monomers 3,6-CB-ODP, CB-TDP, and CB-SDP show an out-of-plane direction comparative with the formation plane, and the electron-giving impacts of oxygen (O), sulfur (S), and selenium (Se) altogether changed. The quadrupole second estimations of CB-TDP and copolymer monomers are bigger than those of CB copolymer monomers, showing that the sulfur (S) iota and selenium (Se) are more grounded electron receptors. The determined band hole E_g_ of the contemplated model mixtures increments all together 3,6-CB-TDP < 3,6-CB-SDP < 3,6-CB-ODP < 3,6-CB-TCP < 3,6-CB-SCP < 3,6-CB-OCP < 3,6-CB-BCT < 3,6-CB-BCS < 3,6-CB-BCO.

A much lower E_g_ of 3,6-CB-TDP and 3,6-CB-SDP than different monomers shows a significant impact of intramolecular burden move, which would cause the assimilation spectra to change red. Subsequently, critical changes were noticed for the optoelectronic properties determined: E_HOMO_, E_LUMO_, E_g_, E_opt_, and E_B_ energies. Additionally, the outcomes likewise propose that various acceptors, such as ODP, TDP, and SDP, may influence the inborn optoelectronic properties of the comparing monomers. The retention properties of UV – Vis were acquired utilizing the TD/DFT/B3LYP/6-311 G technique. The most extreme assimilation got is somewhere in the range of 513 and 666 nm in the gas and dissolvable. The hypothetical photovoltaic estimations of the V_OC_ of the atoms contemplated range from 0.80 to 1.3 eV. Finally, the outcomes acquired show how the electronic properties can be tuned by a substitute with a few gatherings of acceptors and recommend that CB-ODP, CB-TDP, and CB-SDP compounds are acceptable contender for optoelectronic applications like BHJ in the solar cells.
